# Acridine–benzene-1,3,5-tricarb­oxy­lic acid (3/1)

**DOI:** 10.1107/S1600536810050233

**Published:** 2010-12-15

**Authors:** Hossein Aghabozorg, Saba Goodarzi, Masoud Mirzaei, Behrouz Notash

**Affiliations:** a Faculty of Chemistry, Islamic Azad University, North Tehran Branch, Tehran, Iran; b Department of Chemistry, School of Sciences, Ferdowsi University of Mashhad, Mashhad, Iran; c Department of Chemistry, Shahid Beheshti University, G.C., Evin, Tehran 1983963113, Iran

## Abstract

In the title adduct, 3C_13_H_9_N·C_9_H_6_O_6_ or (acr)_3_(btc), associ­ations of one btc and three acr molecules linked by O—H⋯N hydrogen bonds occur. C—H⋯O interactions also occur, resulting in a cyclic hydrogen-bonded synthon *R*_2_^1^(6). The acr mol­ecules and the btc mol­ecules also form slipped or offset π–π stacking inter­actions [centroid–centroid distances of 3.5212 (17) Å for btc rings and 3.703 (2) and 3.731 (2) Å for acr rings]. Together these inter­actions lead to a three-dimensional network.

## Related literature

For background to proton-transfer compounds including acridine, see: Tabatabaee *et al.* (2009 ▶); Eshtiagh-Hosseini *et al.* (2010 ▶). For background to co-crystals, see: Dale *et al.* (2004 ▶). 
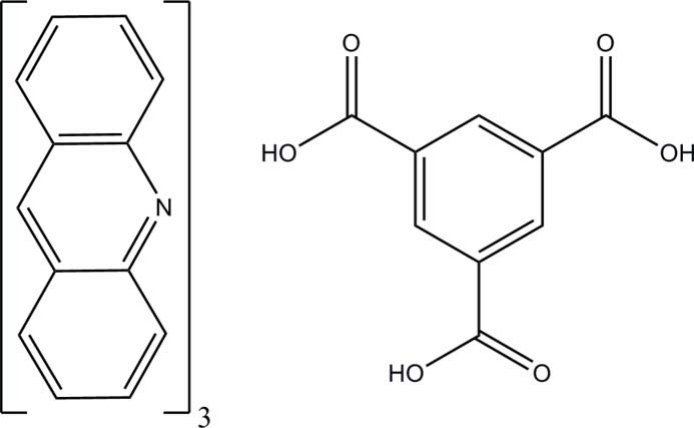


## Experimental

### 

#### Crystal data

3C_13_H_9_N·C_9_H_6_O_6_
*M* *_r_* = 747.77
Triclinic, 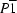

*a* = 12.031 (2) Å
*b* = 13.113 (3) Å
*c* = 13.220 (3) Å
α = 77.44 (3)°
β = 71.43 (3)°
γ = 72.23 (3)°
*V* = 1865.9 (8) Å^3^
*Z* = 2
Mo *K*α radiation
μ = 0.09 mm^−1^
*T* = 298 K
0.45 × 0.3 × 0.2 mm

#### Data collection

Stoe IPDS II diffractometer
Absorption correction: numerical (*X-RED* and *X-SHAPE*; Stoe & Cie, 2005 ▶) *T*_min_ = 0.964, *T*_max_ = 0.980
15233 measured reflections
7305 independent reflections
3826 reflections with *I* > 2σ(*I*)
*R*_int_ = 0.088

#### Refinement

*R*[*F* ^2^ > 2σ(*F* ^2^)] = 0.059
*wR*(*F* ^2^) = 0.218
*S* = 0.95
7305 reflections
526 parameters
H atoms treated by a mixture of independent and constrained refinement
Δρ_max_ = 0.32 e Å^−3^
Δρ_min_ = −0.34 e Å^−3^

### 

Data collection: *X-AREA* (Stoe & Cie, 2005 ▶); cell refinement: *X-AREA*; data reduction: *X-AREA*; program(s) used to solve structure: *SHELXS97* (Sheldrick, 2008 ▶); program(s) used to refine structure: *SHELXL97* (Sheldrick, 2008 ▶); molecular graphics: *ORTEP-3 for Windows* (Farrugia, 1997 ▶); software used to prepare material for publication: *WinGX* (Farrugia, 1999 ▶).

## Supplementary Material

Crystal structure: contains datablocks I, global. DOI: 10.1107/S1600536810050233/om2368sup1.cif

Structure factors: contains datablocks I. DOI: 10.1107/S1600536810050233/om2368Isup2.hkl

Additional supplementary materials:  crystallographic information; 3D view; checkCIF report

## Figures and Tables

**Table 1:** Hydrogen-bond geometry (Å, °)

*D*—H⋯*A*	*D*—H	H⋯*A*	*D*⋯*A*	*D*—H⋯*A*
O1—H1⋯N1	1.03 (4)	1.62 (4)	2.643 (4)	173 (4)
O3—H3⋯N2	1.08 (6)	1.55 (6)	2.619 (4)	166 (5)
O5—H5⋯N3	1.10 (5)	1.57 (5)	2.659 (4)	171 (6)
C14—H14⋯O6^i^	0.93	2.44	3.266 (5)	147
C16—H16⋯O6^i^	0.93	2.55	3.355 (5)	145
C18—H18⋯O2^ii^	0.93	2.54	3.389 (5)	151
C24—H24⋯O5^iii^	0.93	2.53	3.278 (5)	138
C27—H27⋯O4^iv^	0.93	2.59	3.435 (5)	151
C47—H47⋯O3^iii^	0.93	2.56	3.345 (5)	143

Symmetry codes: (i) 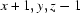; (ii) 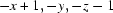; (iii) 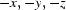; (iv) 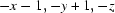.

**Fig. 1.**
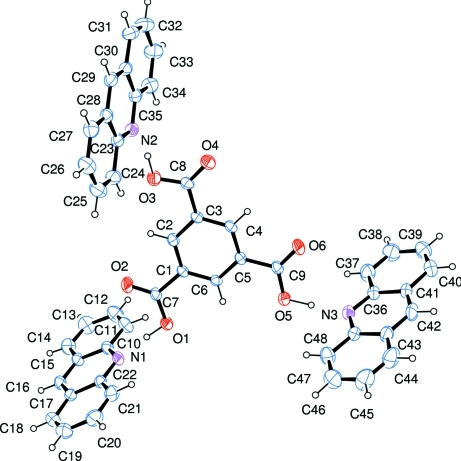
The molecular structure of the title compound with displacement ellipsoids drawn at 30% probability level.

**Fig. 2.**
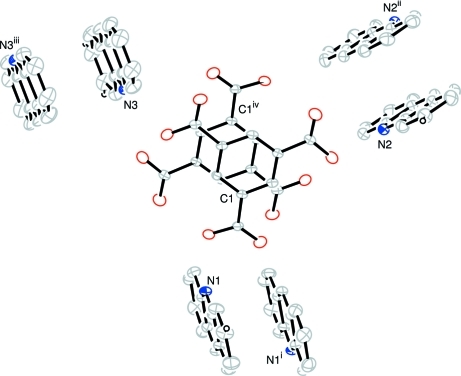
The crystal packing of the title compound along b axis. The figure shows the parallel arrangements between centro-symmetry related aromatic rings [symmetry code: (i) 1-x, -y, -1-z; (ii)-1-x, 1-y, -z; (iii) -x, -1-y, 1-z; (iv) -x,-y,-z].

## supplementary crystallographic information

### Comment

Acridine is structurally related to anthracene wherein one of the central CH group is replaced by nitrogen. It is a raw material used for the production of dyes and some valuable drugs. Our research group has recently reported two proton transfer complexes with acridine (Tabatabaee *et al.*, 2009; Eshtiagh-Hosseini *et al.*, 2010). Recently, Dale *et al.* reported the structure of btc with three pyridines as a cocrystal (Dale *et al.*, 2004). In this article, we report the crystal structure of a new cocrystal system containing acridine and benzenetricarboxylic acid, for the first time.

The title cocrystal structure contains acridine and benzene-1,3,5-tricarboxylic acid in 3:1 molar ratio in the asymmetric unit (Fig. 1). These three bases and one acid formed a cocrystal without any proton transfer. Hence, the acr molecules interact with the carboxylic acid groups of the respective btc molecule through O—H···N and C—H···O hydrogen bonds (Table 1). The latter formed a cyclic hydrogen-bonded synthon *R*^1 ^_2_(6). The acr molecules and also btc molecules form slipped or offset π-π stacking interactions [with centroid···centroid distances of 3.5212 (17) Å for btc rings and 3.703 (2) and 3.731 (2) Å for acr rings]. The dihedral angle of the plane of three carboxylate groups with respect to plane of the central benzene ring in btc are equal to 3.17, 6.46 and 6.52°. Indeed, the crystal structure is stabilized by an extensive series of intermolecular O—H···N and C—H···O hydrogen bonds and π-π stacking interactions, forming a three-dimensional network (Fig. 2).

### Experimental

The reaction between a solution of benzenetricarboxylic acid (70 mg, 0.30 mmol) in 5 ml ethanol and acridine (180 mg, 1.0 mmol) in 5 ml ethanol in 1:3 molar ratio at 298 K for 4 hr gave orange block crystals of (acr)_3_(btc) after slow evaporation of the solvent at room temperature (m.p. > 260 °C).

### Refinement

The hydrogen atoms of the carboxylic part of btc molecule were found in a diference Fourier map and refined isotropically without restraint. All of the other H atoms were positioned geometrically and refined as riding atoms, with C—H = 0.93 and *U*_iso_(H) = 1.2*U*_eq_(C).

### Crystal data

**Table:**

3C_13_H_9_N·C_9_H_6_O_6_	*Z* = 2
*M**_r_* = 747.77	*F*(000) = 780
Triclinic, *P*1	*D*_x_ = 1.331 Mg m^−^^3^
Hall symbol: -P 1	Mo *K*α radiation, λ = 0.71073 Å
*a* = 12.031 (2) Å	Cell parameters from 7305 reflections
*b* = 13.113 (3) Å	θ = 2.1–26.0°
*c* = 13.220 (3) Å	µ = 0.09 mm^−^^1^
α = 77.44 (3)°	*T* = 298 K
β = 71.43 (3)°	Block, orange
γ = 72.23 (3)°	0.45 × 0.3 × 0.2 mm
*V* = 1865.9 (8) Å^3^	

### Data collection

**Table:**

Stoe IPDS II diffractometer	7305 independent reflections
Radiation source: fine-focus sealed tube	3826 reflections with *I* > 2σ(*I*)
graphite	*R*_int_ = 0.088
Detector resolution: 0.15 mm pixels mm^-1^	θ_max_ = 26.0°, θ_min_ = 2.1°
φ scans	*h* = −14→14
Absorption correction: numerical (*X-RED* and *X-SHAPE*; Stoe & Cie, 2005)	*k* = −16→16
*T*_min_ = 0.964, *T*_max_ = 0.980	*l* = −16→15
15233 measured reflections	

### Refinement

**Table:**

Refinement on *F*^2^	Primary atom site location: structure-invariant direct methods
Least-squares matrix: full	Secondary atom site location: difference Fourier map
*R*[*F*^2^ > 2σ(*F*^2^)] = 0.059	Hydrogen site location: inferred from neighbouring sites
*wR*(*F*^2^) = 0.218	H atoms treated by a mixture of independent and constrained refinement
*S* = 0.95	*w* = 1/[σ^2^(*F*_o_^2^) + (0.1227*P*)^2^] where *P* = (*F*_o_^2^ + 2*F*_c_^2^)/3
7305 reflections	(Δ/σ)_max_ = 0.001
526 parameters	Δρ_max_ = 0.32 e Å^−^^3^
0 restraints	Δρ_min_ = −0.34 e Å^−^^3^

### Special details

**Table:**

Geometry. All e.s.d.'s (except the e.s.d. in the dihedral angle between two l.s. planes) are estimated using the full covariance matrix. The cell e.s.d.'s are taken into account individually in the estimation of e.s.d.'s in distances, angles and torsion angles; correlations between e.s.d.'s in cell parameters are only used when they are defined by crystal symmetry. An approximate (isotropic) treatment of cell e.s.d.'s is used for estimating e.s.d.'s involving l.s. planes.
Refinement. Refinement of *F*^2^ against ALL reflections. The weighted *R*-factor *wR* and goodness of fit *S* are based on *F*^2^, conventional *R*-factors *R* are based on *F*, with *F* set to zero for negative *F*^2^. The threshold expression of *F*^2^ > σ(*F*^2^) is used only for calculating *R*-factors(gt) *etc*. and is not relevant to the choice of reflections for refinement. *R*-factors based on *F*^2^ are statistically about twice as large as those based on *F*, and *R*- factors based on ALL data will be even larger.

### Fractional atomic coordinates and isotropic or equivalent isotropic displacement parameters (Å^2^)

**Table:**

	*x*	*y*	*z*	*U*_iso_*/*U*_eq_	
C1	0.1489 (2)	0.0445 (2)	−0.0245 (2)	0.0366 (6)	
C2	0.0715 (3)	0.1475 (3)	−0.0249 (2)	0.0401 (7)	
H2	0.0842	0.1982	−0.0859	0.048*	
C3	−0.0247 (2)	0.1761 (2)	0.0643 (2)	0.0373 (7)	
C4	−0.0432 (3)	0.1001 (3)	0.1548 (2)	0.0401 (7)	
H4	−0.1069	0.1189	0.2151	0.048*	
C5	0.0317 (2)	−0.0032 (2)	0.1564 (2)	0.0373 (7)	
C6	0.1281 (2)	−0.0312 (2)	0.0661 (2)	0.0356 (6)	
H6	0.1786	−0.1009	0.0668	0.043*	
C7	0.2493 (3)	0.0184 (3)	−0.1247 (2)	0.0420 (7)	
C8	−0.1130 (3)	0.2841 (3)	0.0637 (3)	0.0475 (8)	
C9	0.0045 (3)	−0.0833 (3)	0.2553 (2)	0.0441 (7)	
C10	0.5826 (3)	−0.0662 (3)	−0.3125 (2)	0.0487 (8)	
C11	0.5902 (3)	−0.0260 (4)	−0.2253 (3)	0.0663 (11)	
H11	0.5339	−0.0329	−0.1587	0.080*	
C12	0.6791 (4)	0.0227 (4)	−0.2379 (4)	0.0787 (12)	
H12	0.6841	0.0483	−0.1795	0.094*	
C13	0.7643 (4)	0.0348 (4)	−0.3387 (4)	0.0803 (13)	
H13	0.8239	0.0694	−0.3461	0.096*	
C14	0.7611 (3)	−0.0025 (3)	−0.4240 (3)	0.0653 (10)	
H14	0.8181	0.0066	−0.4897	0.078*	
C15	0.6707 (3)	−0.0561 (3)	−0.4144 (3)	0.0483 (8)	
C16	0.6635 (3)	−0.0974 (3)	−0.4987 (3)	0.0517 (9)	
H16	0.7199	−0.0916	−0.5653	0.062*	
C17	0.5721 (3)	−0.1480 (3)	−0.4846 (2)	0.0479 (8)	
C18	0.5598 (4)	−0.1925 (3)	−0.5674 (3)	0.0668 (11)	
H18	0.6151	−0.1898	−0.6350	0.080*	
C19	0.4684 (4)	−0.2389 (4)	−0.5491 (4)	0.0779 (12)	
H19	0.4611	−0.2674	−0.6046	0.094*	
C20	0.3842 (4)	−0.2449 (4)	−0.4480 (4)	0.0741 (12)	
H20	0.3231	−0.2791	−0.4369	0.089*	
C21	0.3901 (3)	−0.2020 (3)	−0.3663 (3)	0.0641 (10)	
H21	0.3317	−0.2043	−0.3004	0.077*	
C22	0.4859 (3)	−0.1532 (3)	−0.3814 (2)	0.0466 (8)	
C23	−0.3427 (3)	0.4865 (3)	−0.1242 (3)	0.0475 (8)	
C24	−0.2966 (3)	0.3906 (3)	−0.1741 (3)	0.0592 (9)	
H24	−0.2266	0.3405	−0.1626	0.071*	
C25	−0.3548 (4)	0.3724 (4)	−0.2383 (3)	0.0709 (11)	
H25	−0.3227	0.3106	−0.2724	0.085*	
C26	−0.4632 (4)	0.4455 (4)	−0.2545 (3)	0.0705 (11)	
H26	−0.5022	0.4309	−0.2981	0.085*	
C27	−0.5107 (3)	0.5364 (3)	−0.2070 (3)	0.0610 (10)	
H27	−0.5827	0.5837	−0.2176	0.073*	
C28	−0.4514 (3)	0.5604 (3)	−0.1410 (3)	0.0494 (8)	
C29	−0.4945 (3)	0.6528 (3)	−0.0911 (3)	0.0523 (8)	
H29	−0.5658	0.7025	−0.1000	0.063*	
C30	−0.4323 (3)	0.6719 (3)	−0.0280 (3)	0.0509 (8)	
C31	−0.4709 (4)	0.7652 (3)	0.0253 (3)	0.0649 (10)	
H31	−0.5416	0.8172	0.0183	0.078*	
C32	−0.4067 (4)	0.7791 (4)	0.0856 (4)	0.0781 (13)	
H32	−0.4333	0.8404	0.1201	0.094*	
C33	−0.2995 (4)	0.7015 (4)	0.0969 (4)	0.0741 (12)	
H33	−0.2558	0.7130	0.1383	0.089*	
C34	−0.2582 (3)	0.6102 (3)	0.0489 (3)	0.0643 (10)	
H34	−0.1877	0.5593	0.0583	0.077*	
C35	−0.3234 (3)	0.5934 (3)	−0.0155 (3)	0.0491 (8)	
C36	−0.0443 (3)	−0.2948 (3)	0.5167 (3)	0.0533 (9)	
C37	0.0199 (4)	−0.2314 (4)	0.5384 (3)	0.0764 (12)	
H37	0.0789	−0.2055	0.4830	0.092*	
C38	−0.0040 (5)	−0.2087 (4)	0.6387 (4)	0.0904 (15)	
H38	0.0384	−0.1664	0.6514	0.108*	
C39	−0.0916 (6)	−0.2472 (4)	0.7246 (4)	0.0945 (16)	
H39	−0.1058	−0.2311	0.7935	0.113*	
C40	−0.1547 (5)	−0.3071 (4)	0.7077 (3)	0.0828 (14)	
H40	−0.2131	−0.3316	0.7650	0.099*	
C41	−0.1338 (4)	−0.3341 (3)	0.6033 (3)	0.0595 (10)	
C42	−0.1932 (4)	−0.3969 (3)	0.5801 (3)	0.0737 (12)	
H42	−0.2517	−0.4239	0.6351	0.088*	
C43	−0.1683 (4)	−0.4208 (3)	0.4771 (3)	0.0607 (10)	
C44	−0.2255 (5)	−0.4863 (4)	0.4478 (4)	0.0911 (15)	
H44	−0.2853	−0.5145	0.4999	0.109*	
C45	−0.1941 (6)	−0.5077 (4)	0.3462 (4)	0.0977 (17)	
H45	−0.2323	−0.5506	0.3285	0.117*	
C46	−0.1043 (5)	−0.4659 (4)	0.2667 (4)	0.0879 (14)	
H46	−0.0807	−0.4843	0.1974	0.106*	
C47	−0.0512 (4)	−0.3994 (4)	0.2888 (3)	0.0697 (11)	
H47	0.0040	−0.3684	0.2338	0.084*	
C48	−0.0789 (3)	−0.3769 (3)	0.3947 (3)	0.0507 (8)	
N1	0.4920 (2)	−0.1125 (2)	−0.2986 (2)	0.0495 (7)	
N2	−0.2812 (2)	0.5027 (2)	−0.0623 (2)	0.0495 (7)	
N3	−0.0211 (3)	−0.3142 (2)	0.4155 (2)	0.0538 (7)	
O1	0.3136 (2)	−0.08203 (19)	−0.12033 (18)	0.0543 (6)	
H1	0.378 (3)	−0.092 (3)	−0.193 (3)	0.063 (10)*	
O2	0.2668 (3)	0.0870 (2)	−0.20116 (19)	0.0759 (9)	
O3	−0.0948 (2)	0.3460 (2)	−0.02961 (19)	0.0640 (7)	
H3	−0.173 (5)	0.414 (5)	−0.032 (4)	0.128 (19)*	
O4	−0.1941 (3)	0.3119 (2)	0.1420 (2)	0.0805 (9)	
O5	0.0707 (2)	−0.1822 (2)	0.24783 (18)	0.0573 (6)	
H5	0.041 (5)	−0.239 (4)	0.319 (4)	0.116 (17)*	
O6	−0.0735 (2)	−0.0553 (2)	0.33509 (19)	0.0731 (8)	

### Atomic displacement parameters (Å^2^)

**Table:**

	*U*^11^	*U*^22^	*U*^33^	*U*^12^	*U*^13^	*U*^23^
C1	0.0283 (14)	0.0458 (17)	0.0357 (14)	−0.0116 (13)	−0.0035 (11)	−0.0106 (12)
C2	0.0337 (15)	0.0490 (19)	0.0381 (15)	−0.0152 (14)	−0.0055 (12)	−0.0061 (13)
C3	0.0296 (14)	0.0428 (17)	0.0389 (15)	−0.0075 (13)	−0.0070 (11)	−0.0101 (13)
C4	0.0311 (14)	0.0497 (19)	0.0384 (15)	−0.0122 (14)	−0.0004 (12)	−0.0144 (13)
C5	0.0313 (14)	0.0481 (18)	0.0349 (14)	−0.0162 (13)	−0.0047 (11)	−0.0080 (12)
C6	0.0289 (13)	0.0354 (16)	0.0398 (15)	−0.0067 (12)	−0.0046 (11)	−0.0088 (12)
C7	0.0345 (15)	0.0481 (19)	0.0378 (15)	−0.0114 (14)	−0.0020 (12)	−0.0050 (14)
C8	0.0370 (16)	0.0484 (19)	0.0491 (18)	−0.0041 (14)	−0.0037 (14)	−0.0119 (15)
C9	0.0385 (16)	0.053 (2)	0.0386 (16)	−0.0161 (15)	−0.0015 (13)	−0.0081 (14)
C10	0.0364 (16)	0.057 (2)	0.0453 (17)	−0.0110 (15)	0.0000 (13)	−0.0088 (15)
C11	0.053 (2)	0.089 (3)	0.054 (2)	−0.018 (2)	−0.0018 (16)	−0.023 (2)
C12	0.067 (3)	0.106 (4)	0.074 (3)	−0.025 (3)	−0.018 (2)	−0.031 (2)
C13	0.061 (3)	0.103 (4)	0.089 (3)	−0.035 (3)	−0.021 (2)	−0.015 (3)
C14	0.0436 (19)	0.081 (3)	0.066 (2)	−0.0226 (19)	−0.0032 (17)	−0.007 (2)
C15	0.0354 (16)	0.053 (2)	0.0468 (18)	−0.0074 (15)	−0.0010 (13)	−0.0073 (15)
C16	0.0401 (17)	0.055 (2)	0.0438 (17)	−0.0079 (16)	0.0042 (14)	−0.0023 (15)
C17	0.0476 (18)	0.0427 (18)	0.0438 (17)	−0.0060 (15)	−0.0047 (14)	−0.0053 (14)
C18	0.076 (3)	0.067 (3)	0.053 (2)	−0.017 (2)	−0.0090 (19)	−0.0157 (18)
C19	0.097 (3)	0.074 (3)	0.073 (3)	−0.029 (3)	−0.023 (2)	−0.021 (2)
C20	0.079 (3)	0.067 (3)	0.088 (3)	−0.035 (2)	−0.024 (2)	−0.006 (2)
C21	0.056 (2)	0.065 (2)	0.065 (2)	−0.0260 (19)	−0.0015 (18)	−0.0029 (19)
C22	0.0426 (17)	0.0456 (18)	0.0438 (17)	−0.0107 (15)	−0.0036 (13)	−0.0032 (14)
C23	0.0431 (17)	0.0467 (19)	0.0439 (17)	−0.0102 (15)	−0.0034 (14)	−0.0027 (14)
C24	0.052 (2)	0.054 (2)	0.061 (2)	−0.0074 (17)	−0.0059 (17)	−0.0097 (17)
C25	0.073 (3)	0.073 (3)	0.066 (2)	−0.025 (2)	−0.002 (2)	−0.024 (2)
C26	0.067 (3)	0.084 (3)	0.066 (2)	−0.025 (2)	−0.011 (2)	−0.022 (2)
C27	0.053 (2)	0.068 (3)	0.059 (2)	−0.0140 (19)	−0.0180 (17)	−0.0020 (19)
C28	0.0446 (17)	0.051 (2)	0.0447 (17)	−0.0136 (16)	−0.0037 (14)	0.0000 (15)
C29	0.0459 (18)	0.049 (2)	0.0515 (18)	−0.0075 (16)	−0.0098 (15)	0.0019 (15)
C30	0.0497 (19)	0.0432 (19)	0.0512 (18)	−0.0106 (16)	−0.0060 (15)	−0.0018 (15)
C31	0.063 (2)	0.047 (2)	0.080 (3)	−0.0069 (18)	−0.018 (2)	−0.0091 (19)
C32	0.081 (3)	0.057 (3)	0.100 (3)	−0.018 (2)	−0.018 (3)	−0.027 (2)
C33	0.074 (3)	0.069 (3)	0.091 (3)	−0.025 (2)	−0.024 (2)	−0.022 (2)
C34	0.054 (2)	0.063 (3)	0.077 (2)	−0.0126 (19)	−0.0213 (19)	−0.009 (2)
C35	0.0477 (18)	0.049 (2)	0.0477 (18)	−0.0162 (16)	−0.0076 (14)	−0.0026 (15)
C36	0.064 (2)	0.052 (2)	0.0419 (17)	−0.0191 (18)	−0.0093 (15)	−0.0019 (15)
C37	0.095 (3)	0.081 (3)	0.066 (3)	−0.045 (3)	−0.019 (2)	−0.005 (2)
C38	0.124 (4)	0.090 (4)	0.077 (3)	−0.043 (3)	−0.035 (3)	−0.019 (3)
C39	0.141 (5)	0.093 (4)	0.055 (2)	−0.032 (4)	−0.025 (3)	−0.019 (2)
C40	0.111 (4)	0.079 (3)	0.048 (2)	−0.030 (3)	−0.004 (2)	−0.004 (2)
C41	0.077 (2)	0.053 (2)	0.0423 (18)	−0.025 (2)	−0.0042 (17)	0.0015 (15)
C42	0.086 (3)	0.075 (3)	0.055 (2)	−0.045 (2)	0.0024 (19)	0.0052 (19)
C43	0.070 (2)	0.057 (2)	0.057 (2)	−0.028 (2)	−0.0150 (18)	0.0029 (17)
C44	0.108 (4)	0.091 (4)	0.095 (3)	−0.062 (3)	−0.034 (3)	0.010 (3)
C45	0.136 (5)	0.096 (4)	0.098 (4)	−0.064 (4)	−0.062 (4)	0.005 (3)
C46	0.112 (4)	0.100 (4)	0.072 (3)	−0.033 (3)	−0.046 (3)	−0.014 (3)
C47	0.075 (3)	0.085 (3)	0.054 (2)	−0.025 (2)	−0.0218 (19)	−0.006 (2)
C48	0.057 (2)	0.0462 (19)	0.0466 (18)	−0.0124 (16)	−0.0155 (15)	−0.0001 (15)
N1	0.0382 (14)	0.0564 (17)	0.0419 (14)	−0.0114 (13)	0.0045 (11)	−0.0060 (12)
N2	0.0426 (15)	0.0472 (17)	0.0515 (15)	−0.0077 (13)	−0.0094 (12)	−0.0022 (13)
N3	0.0585 (17)	0.0560 (18)	0.0450 (15)	−0.0226 (15)	−0.0069 (13)	−0.0018 (13)
O1	0.0431 (12)	0.0534 (15)	0.0454 (12)	−0.0039 (11)	0.0081 (10)	−0.0060 (10)
O2	0.0754 (17)	0.0670 (17)	0.0472 (13)	−0.0086 (14)	0.0170 (12)	0.0053 (12)
O3	0.0534 (14)	0.0623 (16)	0.0524 (14)	0.0052 (13)	−0.0057 (11)	−0.0006 (12)
O4	0.0723 (17)	0.0653 (18)	0.0621 (16)	0.0051 (14)	0.0163 (14)	−0.0081 (13)
O5	0.0611 (15)	0.0496 (15)	0.0467 (13)	−0.0168 (12)	0.0058 (11)	−0.0040 (11)
O6	0.0686 (16)	0.0710 (18)	0.0470 (13)	−0.0110 (14)	0.0193 (12)	−0.0040 (12)

### Geometric parameters (Å, °)

**Table:**

C1—C2	1.388 (4)	C25—C26	1.411 (6)
C1—C6	1.389 (4)	C25—H25	0.9300
C1—C7	1.501 (4)	C26—C27	1.350 (6)
C2—C3	1.389 (4)	C26—H26	0.9300
C2—H2	0.9300	C27—C28	1.422 (5)
C3—C4	1.386 (4)	C27—H27	0.9300
C3—C8	1.488 (4)	C28—C29	1.386 (5)
C4—C5	1.379 (4)	C29—C30	1.386 (5)
C4—H4	0.9300	C29—H29	0.9300
C5—C6	1.397 (4)	C30—C31	1.424 (5)
C5—C9	1.503 (4)	C30—C35	1.430 (5)
C6—H6	0.9300	C31—C32	1.345 (6)
C7—O2	1.208 (4)	C31—H31	0.9300
C7—O1	1.308 (4)	C32—C33	1.405 (6)
C8—O4	1.208 (4)	C32—H32	0.9300
C8—O3	1.316 (4)	C33—C34	1.360 (6)
C9—O6	1.207 (4)	C33—H33	0.9300
C9—O5	1.304 (4)	C34—C35	1.414 (5)
C10—N1	1.349 (4)	C34—H34	0.9300
C10—C11	1.406 (5)	C35—N2	1.344 (4)
C10—C15	1.433 (4)	C36—N3	1.341 (4)
C11—C12	1.356 (6)	C36—C37	1.416 (5)
C11—H11	0.9300	C36—C41	1.426 (5)
C12—C13	1.411 (6)	C37—C38	1.344 (6)
C12—H12	0.9300	C37—H37	0.9300
C13—C14	1.337 (6)	C38—C39	1.403 (7)
C13—H13	0.9300	C38—H38	0.9300
C14—C15	1.427 (5)	C39—C40	1.339 (7)
C14—H14	0.9300	C39—H39	0.9300
C15—C16	1.377 (5)	C40—C41	1.425 (6)
C16—C17	1.395 (5)	C40—H40	0.9300
C16—H16	0.9300	C41—C42	1.376 (6)
C17—C18	1.412 (5)	C42—C43	1.382 (5)
C17—C22	1.430 (4)	C42—H42	0.9300
C18—C19	1.346 (6)	C43—C48	1.426 (5)
C18—H18	0.9300	C43—C44	1.427 (6)
C19—C20	1.400 (6)	C44—C45	1.343 (7)
C19—H19	0.9300	C44—H44	0.9300
C20—C21	1.351 (6)	C45—C46	1.401 (7)
C20—H20	0.9300	C45—H45	0.9300
C21—C22	1.424 (5)	C46—C47	1.350 (6)
C21—H21	0.9300	C46—H46	0.9300
C22—N1	1.348 (4)	C47—C48	1.407 (5)
C23—N2	1.348 (4)	C47—H47	0.9300
C23—C28	1.420 (5)	C48—N3	1.344 (4)
C23—C24	1.423 (5)	O1—H1	1.03 (4)
C24—C25	1.355 (6)	O3—H3	1.08 (6)
C24—H24	0.9300	O5—H5	1.10 (5)
			
C2—C1—C6	119.1 (2)	C27—C26—C25	120.5 (4)
C2—C1—C7	118.1 (3)	C27—C26—H26	119.7
C6—C1—C7	122.7 (3)	C25—C26—H26	119.7
C1—C2—C3	121.0 (3)	C26—C27—C28	120.4 (4)
C1—C2—H2	119.5	C26—C27—H27	119.8
C3—C2—H2	119.5	C28—C27—H27	119.8
C4—C3—C2	119.2 (3)	C29—C28—C23	117.6 (3)
C4—C3—C8	118.7 (2)	C29—C28—C27	123.3 (3)
C2—C3—C8	122.0 (3)	C23—C28—C27	119.1 (3)
C5—C4—C3	120.8 (3)	C28—C29—C30	120.6 (3)
C5—C4—H4	119.6	C28—C29—H29	119.7
C3—C4—H4	119.6	C30—C29—H29	119.7
C4—C5—C6	119.6 (3)	C29—C30—C31	123.7 (3)
C4—C5—C9	118.3 (2)	C29—C30—C35	118.2 (3)
C6—C5—C9	122.0 (3)	C31—C30—C35	118.1 (3)
C1—C6—C5	120.3 (3)	C32—C31—C30	120.9 (4)
C1—C6—H6	119.8	C32—C31—H31	119.6
C5—C6—H6	119.8	C30—C31—H31	119.6
O2—C7—O1	124.5 (3)	C31—C32—C33	120.4 (4)
O2—C7—C1	121.0 (3)	C31—C32—H32	119.8
O1—C7—C1	114.5 (3)	C33—C32—H32	119.8
O4—C8—O3	123.3 (3)	C34—C33—C32	121.6 (4)
O4—C8—C3	122.9 (3)	C34—C33—H33	119.2
O3—C8—C3	113.9 (3)	C32—C33—H33	119.2
O6—C9—O5	123.8 (3)	C33—C34—C35	119.4 (4)
O6—C9—C5	120.8 (3)	C33—C34—H34	120.3
O5—C9—C5	115.4 (2)	C35—C34—H34	120.3
N1—C10—C11	119.2 (3)	N2—C35—C34	118.6 (3)
N1—C10—C15	121.6 (3)	N2—C35—C30	121.9 (3)
C11—C10—C15	119.3 (3)	C34—C35—C30	119.5 (3)
C12—C11—C10	120.3 (3)	N3—C36—C37	119.3 (3)
C12—C11—H11	119.9	N3—C36—C41	122.0 (3)
C10—C11—H11	119.9	C37—C36—C41	118.6 (3)
C11—C12—C13	120.6 (4)	C38—C37—C36	120.3 (4)
C11—C12—H12	119.7	C38—C37—H37	119.8
C13—C12—H12	119.7	C36—C37—H37	119.8
C14—C13—C12	121.3 (4)	C37—C38—C39	121.5 (5)
C14—C13—H13	119.3	C37—C38—H38	119.2
C12—C13—H13	119.3	C39—C38—H38	119.2
C13—C14—C15	120.3 (3)	C40—C39—C38	120.1 (4)
C13—C14—H14	119.9	C40—C39—H39	119.9
C15—C14—H14	119.9	C38—C39—H39	119.9
C16—C15—C14	123.1 (3)	C39—C40—C41	121.1 (4)
C16—C15—C10	118.6 (3)	C39—C40—H40	119.4
C14—C15—C10	118.3 (3)	C41—C40—H40	119.4
C15—C16—C17	120.4 (3)	C42—C41—C40	124.5 (3)
C15—C16—H16	119.8	C42—C41—C36	117.3 (3)
C17—C16—H16	119.8	C40—C41—C36	118.2 (4)
C16—C17—C18	123.3 (3)	C41—C42—C43	121.7 (3)
C16—C17—C22	117.8 (3)	C41—C42—H42	119.1
C18—C17—C22	118.8 (3)	C43—C42—H42	119.1
C19—C18—C17	120.4 (4)	C42—C43—C48	117.4 (3)
C19—C18—H18	119.8	C42—C43—C44	124.6 (4)
C17—C18—H18	119.8	C48—C43—C44	118.0 (4)
C18—C19—C20	121.1 (4)	C45—C44—C43	120.9 (4)
C18—C19—H19	119.4	C45—C44—H44	119.5
C20—C19—H19	119.4	C43—C44—H44	119.5
C21—C20—C19	121.1 (4)	C44—C45—C46	120.4 (4)
C21—C20—H20	119.4	C44—C45—H45	119.8
C19—C20—H20	119.4	C46—C45—H45	119.8
C20—C21—C22	119.8 (3)	C47—C46—C45	121.1 (4)
C20—C21—H21	120.1	C47—C46—H46	119.4
C22—C21—H21	120.1	C45—C46—H46	119.4
N1—C22—C21	119.2 (3)	C46—C47—C48	120.4 (4)
N1—C22—C17	122.2 (3)	C46—C47—H47	119.8
C21—C22—C17	118.7 (3)	C48—C47—H47	119.8
N2—C23—C28	122.9 (3)	N3—C48—C47	119.3 (3)
N2—C23—C24	118.3 (3)	N3—C48—C43	121.7 (3)
C28—C23—C24	118.8 (3)	C47—C48—C43	119.0 (3)
C25—C24—C23	120.0 (4)	C22—N1—C10	119.3 (2)
C25—C24—H24	120.0	C35—N2—C23	118.8 (3)
C23—C24—H24	120.0	C36—N3—C48	119.7 (3)
C24—C25—C26	121.2 (4)	C7—O1—H1	108 (2)
C24—C25—H25	119.4	C8—O3—H3	111 (3)
C26—C25—H25	119.4	C9—O5—H5	112 (3)
			
C6—C1—C2—C3	1.0 (4)	N2—C23—C28—C27	−179.6 (3)
C7—C1—C2—C3	178.6 (3)	C24—C23—C28—C27	−0.1 (4)
C1—C2—C3—C4	−0.1 (4)	C26—C27—C28—C29	179.2 (3)
C1—C2—C3—C8	−176.5 (3)	C26—C27—C28—C23	−1.0 (5)
C2—C3—C4—C5	−0.7 (4)	C23—C28—C29—C30	0.5 (4)
C8—C3—C4—C5	175.8 (3)	C27—C28—C29—C30	−179.7 (3)
C3—C4—C5—C6	0.6 (4)	C28—C29—C30—C31	179.8 (3)
C3—C4—C5—C9	−177.8 (3)	C28—C29—C30—C35	−0.4 (4)
C2—C1—C6—C5	−1.2 (4)	C29—C30—C31—C32	179.9 (4)
C7—C1—C6—C5	−178.7 (3)	C35—C30—C31—C32	0.1 (5)
C4—C5—C6—C1	0.4 (4)	C30—C31—C32—C33	0.2 (6)
C9—C5—C6—C1	178.7 (3)	C31—C32—C33—C34	−0.8 (7)
C2—C1—C7—O2	4.4 (5)	C32—C33—C34—C35	1.0 (6)
C6—C1—C7—O2	−178.1 (3)	C33—C34—C35—N2	180.0 (3)
C2—C1—C7—O1	−176.0 (3)	C33—C34—C35—C30	−0.7 (5)
C6—C1—C7—O1	1.6 (4)	C29—C30—C35—N2	−0.3 (4)
C4—C3—C8—O4	6.9 (5)	C31—C30—C35—N2	179.5 (3)
C2—C3—C8—O4	−176.7 (3)	C29—C30—C35—C34	−179.6 (3)
C4—C3—C8—O3	−173.1 (3)	C31—C30—C35—C34	0.1 (5)
C2—C3—C8—O3	3.3 (4)	N3—C36—C37—C38	178.3 (4)
C4—C5—C9—O6	−7.2 (5)	C41—C36—C37—C38	−0.4 (7)
C6—C5—C9—O6	174.5 (3)	C36—C37—C38—C39	0.6 (8)
C4—C5—C9—O5	173.2 (3)	C37—C38—C39—C40	−0.8 (9)
C6—C5—C9—O5	−5.1 (4)	C38—C39—C40—C41	0.7 (8)
N1—C10—C11—C12	−178.8 (4)	C39—C40—C41—C42	178.7 (5)
C15—C10—C11—C12	0.7 (6)	C39—C40—C41—C36	−0.5 (7)
C10—C11—C12—C13	0.7 (7)	N3—C36—C41—C42	2.4 (6)
C11—C12—C13—C14	−1.0 (8)	C37—C36—C41—C42	−178.9 (4)
C12—C13—C14—C15	−0.2 (7)	N3—C36—C41—C40	−178.4 (4)
C13—C14—C15—C16	−179.1 (4)	C37—C36—C41—C40	0.3 (6)
C13—C14—C15—C10	1.5 (6)	C40—C41—C42—C43	−179.6 (4)
N1—C10—C15—C16	−1.7 (5)	C36—C41—C42—C43	−0.4 (6)
C11—C10—C15—C16	178.9 (3)	C41—C42—C43—C48	−0.6 (6)
N1—C10—C15—C14	177.7 (3)	C41—C42—C43—C44	179.2 (4)
C11—C10—C15—C14	−1.8 (5)	C42—C43—C44—C45	−178.5 (5)
C14—C15—C16—C17	−179.3 (3)	C48—C43—C44—C45	1.4 (7)
C10—C15—C16—C17	0.0 (5)	C43—C44—C45—C46	0.0 (9)
C15—C16—C17—C18	−179.8 (3)	C44—C45—C46—C47	−3.1 (9)
C15—C16—C17—C22	1.2 (5)	C45—C46—C47—C48	4.7 (8)
C16—C17—C18—C19	−179.0 (4)	C46—C47—C48—N3	177.2 (4)
C22—C17—C18—C19	0.0 (6)	C46—C47—C48—C43	−3.2 (6)
C17—C18—C19—C20	−0.5 (7)	C42—C43—C48—N3	−0.3 (6)
C18—C19—C20—C21	1.7 (7)	C44—C43—C48—N3	179.8 (4)
C19—C20—C21—C22	−2.4 (7)	C42—C43—C48—C47	−180.0 (4)
C20—C21—C22—N1	−178.8 (4)	C44—C43—C48—C47	0.2 (6)
C20—C21—C22—C17	1.9 (6)	C21—C22—N1—C10	−180.0 (3)
C16—C17—C22—N1	−0.9 (5)	C17—C22—N1—C10	−0.7 (5)
C18—C17—C22—N1	180.0 (3)	C11—C10—N1—C22	−178.6 (3)
C16—C17—C22—C21	178.4 (3)	C15—C10—N1—C22	2.0 (5)
C18—C17—C22—C21	−0.7 (5)	C34—C35—N2—C23	−179.7 (3)
N2—C23—C24—C25	−178.9 (3)	C30—C35—N2—C23	1.0 (4)
C28—C23—C24—C25	1.6 (5)	C28—C23—N2—C35	−1.0 (4)
C23—C24—C25—C26	−2.0 (5)	C24—C23—N2—C35	179.5 (3)
C24—C25—C26—C27	0.9 (6)	C37—C36—N3—C48	178.0 (4)
C25—C26—C27—C28	0.7 (6)	C41—C36—N3—C48	−3.3 (5)
N2—C23—C28—C29	0.2 (4)	C47—C48—N3—C36	−178.1 (3)
C24—C23—C28—C29	179.7 (3)	C43—C48—N3—C36	2.2 (5)

### Hydrogen-bond geometry (Å, °)

**Table:**

*D*—H···*A*	*D*—H	H···*A*	*D*···*A*	*D*—H···*A*
O1—H1···N1	1.03 (4)	1.62 (4)	2.643 (4)	173 (4)
O3—H3···N2	1.08 (6)	1.55 (6)	2.619 (4)	166 (5)
O5—H5···N3	1.10 (5)	1.57 (5)	2.659 (4)	171 (6)
C14—H14···O6^i^	0.93	2.44	3.266 (5)	147
C16—H16···O6^i^	0.93	2.55	3.355 (5)	145
C18—H18···O2^ii^	0.93	2.54	3.389 (5)	151
C24—H24···O5^iii^	0.93	2.53	3.278 (5)	138
C27—H27···O4^iv^	0.93	2.59	3.435 (5)	151
C47—H47···O3^iii^	0.93	2.56	3.345 (5)	143

Symmetry codes: (i) *x*+1, *y*, *z*−1; (ii) −*x*+1, −*y*, −*z*−1; (iii) −*x*, −*y*, −*z*; (iv) −*x*−1, −*y*+1, −*z*.
